# COVID-19 pandemic: Prevalence of depression, anxiety, and stress symptoms among Brazilian psychologists

**DOI:** 10.3389/fpsyg.2022.1012543

**Published:** 2022-12-02

**Authors:** Juliana Alvares Duarte Bonini Campos, Lucas Arrais Campos, Bianca Gonzalez Martins, Adriano Palomino de Oliveira, Fabiana Maria Navarro, Simone Cristina dos Santos, Josilene da Costa, Oliver Zancul Prado, João Marôco

**Affiliations:** ^1^Department of Biological Sciences, School of Pharmaceutical Sciences, São Paulo State University (UNESP), Araraquara, Brazil; ^2^ Psychology department, Universidade Paulista – UNIP, Araraquara, Brazil; ^3^Faculty of Medicine and Health Technology, Tampere University, Tampere, Finland; ^4^Tampere University Hospital, Tampere, Finland; ^5^Pediatric Dentistry and Orthodontics Department, School of Dentistry, São Paulo State University (UNESP), Araraquara, Brazil; ^6^William James Center for Research (WJCR), University Institute of Psychological, Social, and Life Sciences (ISPA), Lisbon, Portugal

**Keywords:** mental health, pandemic, psychology, anxiety, stress

## Abstract

**Introduction:**

Symptoms related to mental health disorders became the background of the COVID-19 pandemic, and psychologists had to adapt to the demands, while they themselves were exposed to the pandemic and its stressors.

**Objectives:**

To identify demographic and professional characteristics of Brazilian psychologists in different phases of pandemic and their reported care practices, concerns, and symptoms.

**Methods:**

This was an observational study conducted online in four independent phases with no pairing among the samples (May/June 2020, *n* = 263; November/December 2020, *n* = 131; May/June 2021, *n* = 378; November/December 2021, *n* = 222). Depression, Anxiety and Stress Scale was used. The validity and reliability of the data obtained with the DASS-21 were attested to by confirmatory factor analysis. Basic lexographic and similarity analysis were conducted to obtain textual information. Prevalence of variables was estimated and compared between phases using the *z*-test (α = 5%). Similarity analysis was performed to identify the psychologists’ concerns.

**Results:**

Most of participants were women and were self-employed or employed. There was rapid adjustment to remote work and more than 70% reported changes in their mental health since the onset of pandemic. One in four participants had a previous mental health disorder, and there was a high prevalence of symptoms such as anxiety, fear, and angst. The prevalence of professionals who reported not caring about their own mental health was significant. In 2020, one cluster (health) of concern was identified, while in 2021 there were three clusters (health, family, and COVID-19). The prevalence of depression, anxiety, and stress symptoms was high and did not change during the pandemic.

**Conclusions:**

Psychologists adapted to the demands of the population in the face of the pandemic. However, there was a high prevalence of mental health symptoms and a disregard for self-care among these professionals.

## Introduction

The COVID-19 pandemic disrupted routines and forced populations to rapidly adapt to a new and challenging context (Brooks et al., 2020; Cullen et al., 2020; Salari et al., 2020). Mental health care needs and emergencies have increased, both because of challenges directly related to the pandemic itself and because of actions to contain the spread of the virus (such as social distancing and hygiene habits), which has been extensively documented in the literature since previous pandemics (Blendon et al., 2004; Cava et al., 2005; Lee et al., 2005; Wang et al., 2011). Huremovic (2019) and Taylor (2019) published reports of mental health disorders symptoms resulting from various health crises shortly before the outbreak of COVID-19. Among the aspects pointed out by the authors is the presence of emotional and social distress with an increase in anxiety, depression, and stress symptoms, which are directly related to the sudden changes in routine, unpredictability and lack of control of the event and life itself, fear of infection of oneself or family members, stigmatization, and awareness of finitude from the increased death rate (Taylor, 2019; Campos et al., 2020; Faro et al., 2020; Salari et al., 2020; Vindegaard and Benros, 2020; Wang et al., 2020).

Crepaldi et al. (2020) summarized the experiences reported during the COVID-19 pandemic and identified new psychological demands, primarily due to hospital bans on visits, multiple deaths within the same family, and changes in death rituals that significantly complicate the grieving process. This context significantly affected the job of psychologists in hospitals, private clinics, public healthcare, and referral centers, as all contexts of people’s lives were directly or indirectly affected. Changes were necessary to minimize the impact of mental health on the population and to act in an educational, preventive, and interventional manner. For example, professional practice regulations were revised (Conselho Federal de Psicologia [CFP], 2018, 2020), and remote (online) psychosocial support was approved and implemented with the goal of reducing stress and distress and prevent future disorders from the pandemic (Danzmann et al., 2020; Marasca et al., 2020; Noal et al., 2020; Zwielewski et al., 2020).

A study by Campos et al. (2021b) from the beginning of the pandemic found that among health professionals in Brazil, psychologists were the most willing to adopt remote therapy (64.0%). This adaptation was an important step for both the expansion and continuity of care, considering the social distancing measures. However, this change required new strategies to establish an effective therapist-patient relationship and deliver online psychotherapy (Faria, 2019; Danzmann et al., 2020), which presented these professionals with new challenges (Shojaei and Masoumi, 2020). Although more and more data on the mental health of health professionals have emerged since the outbreak of the pandemic (Pappa et al., 2020; Sheraton et al., 2020; Vizheh et al., 2020; Hao et al., 2021; Saragih et al., 2021), not enough has been found on the mental health of psychologists. Generally, these studies focus on information from the medical and nursing fields, while psychologists are usually placed in the broader category of “other professions.” In addition, most studies were conducted in hospitals (Hao et al., 2021).

Campos et al. (2021b) found that the immediate psychological impact of the pandemic was lower among psychologists than other professionals such as dentists, pharmacists, and nutritionists, which was attributed to their training and ability to develop better coping strategies. However, the authors note that this fact does not protect psychologists from the effects of the pandemic on their mental health, as noted by the high prevalence of depressive, anxiety, and stress symptoms among them. Thus, a follow-up study may provide data on psychologists’ mental health during the pandemic, which is still ongoing, and online psychotherapy is being consolidated and becoming a new professional routine. Crescenzo et al. (2021a,b) observed a prevalence of approximately 17.0% of general burnout (high scores of emotional exhaustion and depersonalization and low scores of personal accomplishment) among Italian psychologists during the first wave of COVID-19. The authors suggest that actions and policies aimed at the attention and promotion of occupational health in this professional category are necessary since psychologists have a prominent role in the emergency care of the population since the beginning of the pandemic. Reno-Chanca et al. (2021) investigated the symptoms of anxiety, depression and stress and estimated their impact on the development of obsessions and compulsions in Spanish psychologists, health professionals (non-psychologists), and the general community from July to September 2020. The results were compared and it was found that psychologists had fewer symptoms than the other two groups. It was also found that stress and anxiety were not predictors of compulsion for psychologists. According to the authors (Reno-Chanca et al., 2021), this may suggest that professional background and experience may play a role as a protective factor. Another aspect highlighted is that many psychologists were able to work online during the pandemic while other health professionals required face-to-face contact to offer health services. Face-to-face contact certainly increased the exposure and vulnerability of non-psychologist health professionals to both physical and mental issues, thus justifying the higher anxiety, depression and stress scores among them.

This study was conducted to provide a more detailed overview of the performance and mental health of psychologists that may help develop counseling and support actions. The aim of this study was to i. identify the demographic and professional characteristics of Brazilian psychologists during the COVID-19 pandemic; ii. assess the health practices and symptoms reported by psychologists and compare them at different phases of the pandemic; and iii. identify the main concerns of psychologists during the pandemic.

## Materials and Methods

### Study design and participants

This was a cross-sectional observational study with online data collection conducted in four independent phases,^1^ i.e., there was no pairing among the samples (participants in the first phase did not necessarily participate in the other phases). Participants were psychologists working in different Brazilian states. A non-probability snowball method was used for recruitment. Data were collected online through the Google Forms platform (phases 1 and 2) and Lime Survey (phases 3 and 4). The regional psychology councils of all Brazilian states were first contacted by email and asked to send the research link to the registered professionals. Psychology schools (public and private) were also contacted by email and asked to distribute the link to the survey. All contacts were obtained from the official websites of the councils or universities. Since we used a psychometric scale for identifying symptoms of depression, anxiety and stress (DASS-21, described below), the minimum sample size was calculated considering the need of 5 to 10 persons per item (Hair et al., 2019). Thus, the minimum sample size should be 105 to 210 participants.

Information was collected on age (years), gender (male, female, non-binary, not informed), state of residency, monthly family income (range), type of health care (none, SUS, private insurance, private doctor), income since pandemic began (none, decreased, stable, increased), current employment (retired, unemployed, employed with a formal contract, self-employed), working status since the start of the pandemic (stopped working; continued in-person work; in-person work but with adjustments; remote work; remote and in-person work), and being a frontline worker for COVID-19 (no, yes). Questions were also asked about the pandemic: ‘do you think the coronavirus is dangerous?’ (no, yes), ‘do you think social distancing is important?’ (no, yes), ‘are you in social isolation?’ (no, yes), ‘what do you think of the news?’ (very confusing, confusing, adequate, adequate and informative, adequate and very informative), ‘how do you classify your social life since the pandemic began?’ (very unsatisfactory, unsatisfactory, normal, satisfactory, very satisfactory), ‘how do you feel about the current scenario of the pandemic?’ (very uncertain, uncertain, certain, very certain), ‘do you know anyone who’s tested positive for COVID-19?’ (no, yes), and ‘have you tested positive for COVID-19?’ (no, yes). Participants were also asked if they have ever been diagnosed with a mental disorder before the pandemic, and if yes, what the diagnosis was, if there were changes in their mental health status since the pandemic began, what were the specific symptoms, if mental health was cared for (no, yes) and how (medication, therapy, lifestyle (strategy used), and others; more than one category could be selected). The participant was also asked to name their top three concerns about the pandemic.

### Measuring scale

The Depression, Anxiety and Stress Scale (DASS) developed by Lovibond and Lovibond (1995) was used. The reduced scale has 21 items to assess different aspects of depression (items 3, 5, 10, 13, 16, 17 and 21), anxiety (items 2, 4, 7, 9, 15, 19, 20), and stress (items 1, 6, 8, 11, 12, 14, 18). The responses have a 4-point Likert-type scale from 0 to 3 (0 – never, not applied at all; 1 – sometimes, applied to some degree, or for some time; 2 – very often, used sometimes to a considerable degree, or for a good part of the time; 3 – almost always, applied a lot, or most of the time). The Portuguese version used in the present study was adapted from Vignola and Tucci (2014) by Martins et al. (2019).

### Psychometric indicators

The psychometric indicators of the DASS-21 were evaluated to confirm the validity and reliability of the data. Factor validity was estimated using confirmatory factor analysis (CFA) with the robust weighted least squares adjusted for mean and variance (WLSMV) estimation method. The fit of the model was assessed using the Comparative Fit Index (CFI), the Tucker-Lewis Index (TLI), and the Root Mean Square Error of Approximation (RMSEA) with 90% confidence interval. The model fit was considered reasonable, as CFI and TLI ≥ 0.90 and RMSEA ≤0.10 (Hair et al., 2019; Marôco, 2021). The data had reasonable reliability (internal consistency) based on the ordinal alpha coefficient > 0.80 (Table 1). The MPLUS 8.3 program (Muthén and Muthén, 1998–2017) (Muthén and Muthén, Los Angeles, CA) was used for the analyses.

**Table 1:** Psychometric indicators of depression, anxiety and stress scale (DASS-21) fitted for the samples.

		Confirmatory factor analyses			
Sample	*n*	CFI	TLI	RMSEA]CI90%[	α
Phase 1	263	0.97	0.97	0.064]0.055–0.073[	0.89–0.94
Phase 2	131	0.96	0.96	0.070]0.055–0.084[	0.82–0.94
Phase 3	378	0.98	0.98	0.057]0.050–0.064[	0.90–0.94
Phase 4	222	0.98	0.98	0.053]0.042–0.064[	0.88–0.95

CFI, comparative fit index; TLI, Tuker-Lewis index; RMSEA]CI90%[, root mean square error of approximation with 90% confidence interval; α, ordinal alpha coefficient.

### Statistical analysis

Descriptive statistics were performed to characterize the sample. The prevalence of depressive, anxiety, and stress symptoms (DASS-21) in the sample was estimated for each phase of the study. The cutoff points suggested by Lovibond and Lovibond (1995) were used to categorize participants by level of symptoms using the sum of responses for each DASS factor multiplied by two (Depression: Normal 0 to 9, Mild 10 to 13, Moderate 14 to 20, Severe 21 to 27, and Extremely Severe ≥28; Anxiety: Normal 0 to 7, Mild 8 to 9, Moderate 10 to 14, Severe 15 to 19, and Extremely Severe ≥20; Stress: Normal 0 to 14, Mild 15 to 18, Moderate 19 to 25, Severe 26 to 33, and Extremely Severe ≥34). Prevalence of level of symptoms, mental health care, and lifestyle strategies in the different phases of the study was compared using the z-test and a significance level (α) of 5%.

### Lexical and similarity analysis

Analysis of the psychologists’ main concerns was performed using basic lexical analysis and similarity analysis, considering the grouped information for the year 2020 (phases 1 and 2) and 2021 (phases 3 and 4). In the basic lexical analysis, the number of text segments analyzed, the number of occurrences, the number of forms, and the hapax count (words that appear only once in relation to the total number of words (occurrences) and the total number of forms) was estimated. A word cloud was also created, ranking the words according to their frequency.

The similarity analysis is based on graph theory and indicates the frequency and relationship among professionals’ concerns. The results are presented using a static graph with a Fruchterman and Reingold (1991) representation constructed with the program Interface de R pour the analysis Multidimensionnelles de Textes et de Questionnaires – Iramuteq® version 0.7 alpha 2 (Ratinaud, Déjean and Skalinder, Laboratoire LERASS, Université Tolouse, France, 2008–2014).

### Ethical aspects

Participants voluntarily accessed the link to the survey and signed the informed consent form. The study followed the ethical guidelines of the National Health Council Decision 466/12 and 510/2016 and the guidelines of resolution No. 1/2021-CONEP/SECNS/MS on research in a virtual environment. This study was approved by the National Research Ethics Committee of the Ministry of Health (CONEP) (CAAE 30604220.4.0000.0008).

## Results

Table 2 shows the characteristics of the participants in the different phases of the study. Although the samples presented statistically significant differences in their characteristics, a low effect size can be noted, i.e., these differences have little practical effect. The only characteristic with a substantial difference was the one related to work during the pandemic (Did you keep working during the pandemic?). However, this difference was expected because the data were collected at different times during the pandemic. Overall, there was a greater participation of women (83.7–93.9%), of people from the southeastern region (46.0–65.6%), people whose monthly family income was in the middle class (40.5–53.2%), and people with a private health insurance (65.7–83.2%). The most common work statuses were self-employed (37.1–43.4%) and employed with a formal contract (46.3–49.6%). Fifteen percent of psychologists stopped working at the beginning of the pandemic and this number decreased as the pandemic progressed. A rapid adaptation to remote work occurred throughout the pandemic and was the most common work model (30.4–63.9%), followed by a hybrid model (27.5–38.8%). Most participants did not work on the frontlines of the pandemic (85.1–93.1%).

**Table 2:** General characteristics of participants in the different phases of the study.

**Characteristic**	**Phase 1**	**Phase 2**	**Phase 3**	**Phase 4**	**Total sample**	***p*-value**	Effect size*
***n***	263	131	378	222	994		
**Age** mean ± standard deviation	37.6 ± 12.1^a^	40.1 ± 13.5^a,b^	40.6 ± 13.1^b^	40.8 ± 12.8^b^	39.8 ± 12.8	0.013	0.011
**Sex**	***n* (%)**						
Male	43 (16.3)^a^	8 (6.1)^b^	54 (14.3)^a^	28 (12.6)^a^	133 (13.4)		
Female	220 (83.7)^a^	123 (93.9)^b^	323 (85.4)^a^	191 (86.0)^a^	857 (86.2)	0.040	0.092
Non-binary	-	-	1 (0.3)	2 (0.9)	3 (0.3)		
Not informed	-	-	-	1 (0.5)	1 (0.1)		
**Brazil region**							
North	27 (10.3)^b^	3 (2.3)^a^	17 (4.5)^a^	10 (4.5)^a^	57 (5.8)		
North East	59 (22.4) ^b^	16 (12.2) ^a^	60 (15.9)^a^	30 (13.5) ^a^	165 (16.6)		
Midwest	22 (8.4)^a^	9 (6.9)^a^	19 (5.0)^a^	12 (5.4)^a^	62 (6.2)		
Southeast	121 (46.0)^b^	86 (65.6)^a^	223 (59.2)^a^	138 (62.2)^a^	568 (57.2)		
South	34 (12.9)^a^	17 (13.0)^a^	58 (15.4)^a^	32 (14.4)^a^	141 (14.2)	0.001	0.108
**Health insurance**							
None	11 (4.2)^b^	2 (1.5)^a^	7 (1.9)^a^	3 (1.4)^a^	23 (2.3)		
SUS (UPA)	72 (27.4)^c^	19 (14.5)^a^	79 (20.9)^b^	51 (23.0)^b^	221 (22.3)		
Private health insurance	173 (65.7)^c^	109 (83.2)^a^	286 (75.6)^b^	163 (73.3)^b^	731 (73.5)		
Private physician	7 (2.7)^a^	1 (0.8)^a^	6 (1.6)^a^	5 (2.3)^a^	19 (1.9)	0.028	0.079
**Monthly family income (R$)**							
<1,255.00	15 (5.7)	3 (2.3)	7 (1.9)	8 (3.6)	33 (3.3)		
1,255.00 ┤2,004.00	21 (8.0)	8 (6.1)	28 (7.4)	4 (6.3)	71 (7.2)		
2,004.00 ┤8,640.00	140 (53.2)	53 (40.5)	173 (45.8)	98 (44.2)	464 (46.9)		
8,640.00┤11,261.00	43 (16.3)	31 (23.7)	86 (22.8)	44 (19.8)	204 (20.6)		
>11,261.00	44 (16.8)	32 (24.4)	84 (22.1)	58 (26.1)	218 (22.0)	0.048	0.005
**Working status (current)**							
Retired	…	10 (8.6)	24 (7.0)	10 (4.9)	44 (6.6)		
Unemployed	…	6 (5.2)	15 (4.3)	11 (5.4)	32 (4.8)		
Employee (formal)	…	57 (49.1)	171 (49.6)	95 (46.3)	323 (48.5)		
Self-employed	…	43 (37.1)	135 (39.1)	89 (43.4)	267 (40.1)	0.781	-
**Did you keep working during the pandemic?**							
No	41 (15.6)	8 (6.1)	35 (9.3)	5 (2.5)	89 (9.2)		
Yes, in-person as usual	14 (5.3)	10 (7.6)	35 (9.3)	29 (14.4)	88 (9.0)		
Yes, in-person but my routine has been adapted	40 (15.2)	15 (11.5)	53 (14.0)	28 (13.9)	136 (14.0)		
Yes, remotely	168 (63.9)	62 (47.3)	154 (40.7)	61 (30.4)	445 (45.7)		
Yes, partly in-person, partly remotely	…	36 (27.5)	101 (26.7)	78 (38.8)	215 (22.1)	<0.001	0.230
**Do you work at the frontline in the fight against Covid-19?**							
No	…	122 (93.1)	338 (89.4)	189 (85.1)	649 (88.8)		
Yes	…	9 (6.9)	40 (10.6)	33 (14.9)	82 (11.2)	0.061	0.088

Comparison between samples (α = 5%): age: Kruskal-Wallis Test (*partial eta squared); other variables: chi-square test and residuals analyses (*Cramer’s V). ^a,b^Different letters indicate statistical difference.

The majority of participants believed that the coronavirus was dangerous (97.7–98.7%), that social isolation was important (73.4–97.7%), knew someone who had COVID-19 (70.0–99.5%), and had not tested positive for COVID-19 at the time of the survey (79.3–91.6%) (Table 3). Most participants found the news about the pandemic appropriate (51.2–62.7%). Strikingly, a high number of participants rated their social contacts during the pandemic as normal (38.7–45.0%) or unsatisfactory (18.9–35.0%) and felt insecure towards the pandemic scenario (71.6–88.3%). In addition, many participants reported changes in their monthly family income and more than 70% of participants reported a change in their mental health since the start of the pandemic.

**Table 3:** Distribution of participants in categories about information related to the COVID-19 pandemic in the different phases of the study.

**Questions**	**Phase 1**	**Phase 2**	**Phase 3**	**Phase 4**	**Total sample**
**Do you think the coronavirus is dangerous?**					
No	5 (1.9)	3 (2.3)	5 (1.3)	3 (1.4)	16 (1.6)
Yes	258 (98.1)	128 (97.7)	373 (98.7)	219 (98.6)	978 (98.4)
**Do you think social isolation is important right now?**					
No	6 (2.3)	3 (2.3)	15 (4.0)	59 (26.6)	83 (8.4)
Yes	257 (97.7)	128 (97.7)	363 (96.0)	163 (73.4)	911 (91.6)
**Regarding the news about the COVID-19 pandemic, do you believe that they are/have been:**					
Very confusing	21 (8.0)	13 (9.9)	33 (8.7)	12 (5.4)	79 (8.0)
Confusing	77 (29.3)	51 (38.9)	142 (37.6)	71 (32.2)	341 (34.3)
Adequate	70 (26.6)	43 (32.9)	129 (34.1)	73 (33.1)	315 (31.8)
Adequate and informative	69 (26.2)	22 (16.8)	62 (16.4)	53 (23.9)	206 (20.7)
Adequate and very informative	26 (9.9)	2 (1.5)	12 (3.2)	12 (5.4)	2 (5.2)
**Generally speaking, your social contacts (in-person or online) can be considered:**					
Very unsatisfactory	…	5 (3.8)	23 (6.1)	7 (3.2)	35 (4.8)
Unsatisfactory	…	32 (24.4)	109 (28.9)	35 (15.7)	176 (24.1)
Normal	…	53 (40.5)	146 (38.7)	100 (45.0)	299 (41.0)
Satisfactory	…	36 (27.5)	92 (24.4)	71 (32.0)	199 (27.3)
Very satisfactory	…	5 (3.8)	7 (1.9)	9 (4.1)	21 (2.9)
**In the face of the current pandemic scenario, how do you feel?**					
Very uncertain	56 (21.3)	25 (19.1)	75 (19.8)	16 (7.2)	172 (17.3)
Uncertain	159 (60.4)	88 (67.2)	259 (68.5)	143 (64.4)	649 (65.3)
Confident	46 (17.5)	18 (13.7)	43 (11.4)	61 (27.5)	168 (16.9)
Very confident	2 (0.8)	-	1 (0.3)	2 (0.9)	5 (0.5)
**Do you know someone who has tested positive for COVID-19?**					
No	79 (30.0)	4 (3.1)	2 (0.5)	1 (0.5)	86 (8.7)
Yes	184 (70.0)	127 (96.9)	376 (99.5)	221 (99.5)	908 (91.3)
**Have you tested positive for Covid-19?**					
No	…	120 (91.6)	322 (85.2)	176 (79.3)	618 (84.5)
Yes	…	11 (8.4)	56 (14.8)	46 (20.7)	113 (15.5)
**During the pandemic, your monthly household income:**					
I lost completely my income	…	1 (0.8)	5 (1.3)	9 (4.1)	15 (2.1)
Decreased	…	57 (43.5)	119 (31.5)	67 (30.2)	243 (33.2)
Was maintained	…	57 (43.5)	188 (49.7)	99 (44.5)	344 (47.1)
Increased	…	16 (12.2)	66 (17.5)	47 (21.2)	129 (17.6)
**In the context of the pandemic, have you noticed any changes regarding your mental health?**					
No	78 (29.7)	35 (26.7)	97 (25.7)	44 (19.8)	254 (25.6)
Yes	185 (70.3)	96 (73.3)	281 (74.3)	178 (80.2)	740 (74.4)
**Symptom**					
Anxiety	190 (72.2)	101 (77.1)	293 (77.5)	177 (79.7)	761 (76.6)
Anguish	140 (53.2)	82 (62.6)	218 (57.7)	143 (64.4)	583 (58.7)
Shortness of breath	60 (22.8)	23 (17.6)	70 (18.5)	44 (19.8)	197 (19.8)
Fear	129 (49.0)	79 (60.3)	229 (60.6)	140 (63.1)	577 (58.0)
Panic	19 (7.2)	15 (11.5)	34 (9.0)	25 (11.3)	93 (9.4)
Tachycardia	70 (26.6)	33 (25.2)	96 (25.4)	54 (4.3)	253 (25.5)
Insomnia	153 (58.2)	73 (55.7)	205 (54.2)	130 (58.6)	561 (56.4)
Others	22 (8.4)	4 (3.1)	46 (12.2)	28 (12.6)	100 (10.1)
**Previous mental health disorder (diagnosed)**					
Anxiety disorder	43 (16.3)	20 (15.3)	56 (14.8)	33 (14.9)	152 (15.3)
Panic syndrome	5 (1.9)	2 (1.6)	8 (2.1)	7 (3.2)	22 (2.2)
Depressive disorder	46 (17.5)	16 (12.2)	65 (17.2)	33 (14.9)	160 (16.1)
Bipolar disorder	2 (0.8)	2 (1.5)	2 (0.5)	1 (0.5)	7 (0.7)
Phobic disorders	-	1 (0.8)	2 (0.5)	2 (0.9)	5 (0.5)
Obsessive–compulsive disorder	1 (0.4)	1 (0.8)	4 (1.1)	1 (0.5)	7 (0.7)
Others	7 (2.7)	1 (0.8)	10 (2.6)	9 (4.1)	27 (2.7)
**Do you take care of your mental health?**					
No	55 (20.9)	6 (4.6)	49 (13.0)	29 (13.1)	84 (11.5)
Yes	208 (79.1)	125 (95.4)	329 (87.0)	193 (86.9)	647 (88.5)

At least 1 in 4 participants had been affected by a mental disorder before the pandemic (phase 1: *n* = 263, 28.1%, phase 2: *n* = 131, 25.2%, phase 3: *n* = 378, 28.6%, phase 4: *n* = 222, 27.4%; Table 3). Psychologists reported the presence of mental health disorders symptoms since the beginning of the pandemic; in particular, a high prevalence of anxiety, angst, fear, and insomnia was found in all phases. Anxiety and depressive disorders were the most prevalent. In phases 1, 3, and 4, the high prevalence of professionals who reported not taking care of their own mental health was outstanding.

**Figure 1:**
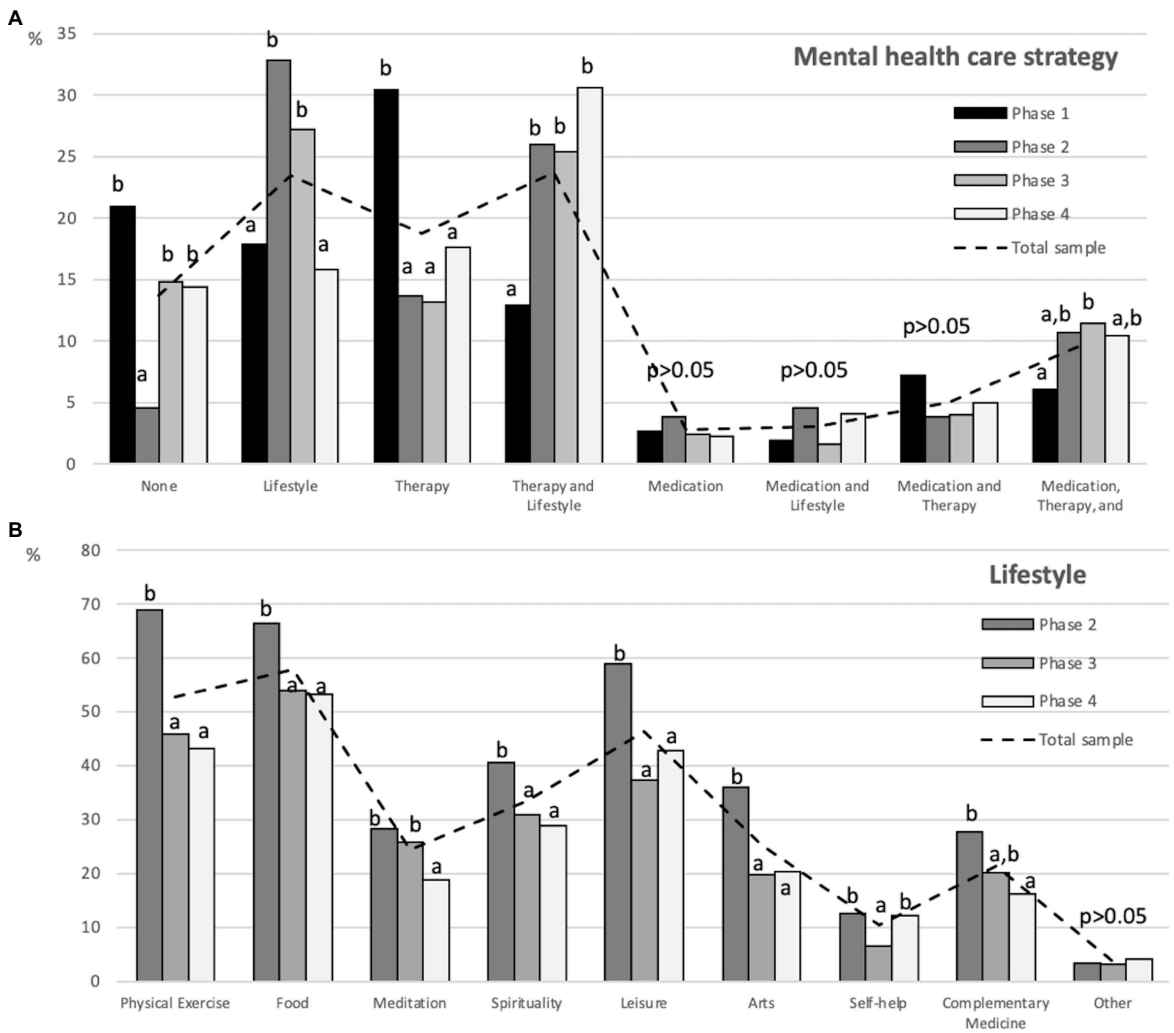
Distribution of participants at each phase of data collection by type of mental health care strategy.

Figure 1 shows the distribution of participants by type of psychosocial support they received and by the strategy they used to achieve a healthier lifestyle. In phase 1, there was a high prevalence of professionals who did not care about their mental health, which decreased in phase 2, but increased again in 2021 (phases 3 and 4). Importance given to lifestyle was higher in phases 2 and 3 of the study, and there was a decrease in the use of therapy alone as mental health care and an increase in the use of therapy combined with lifestyle changes. The use of combined care (medication, therapy, and lifestyle) also increased from the first phase of data collection to the other phases. In general, there was a higher investment in implementing a healthier lifestyle in phase 2, but it decreased in the following phases.

**Figure 2:**
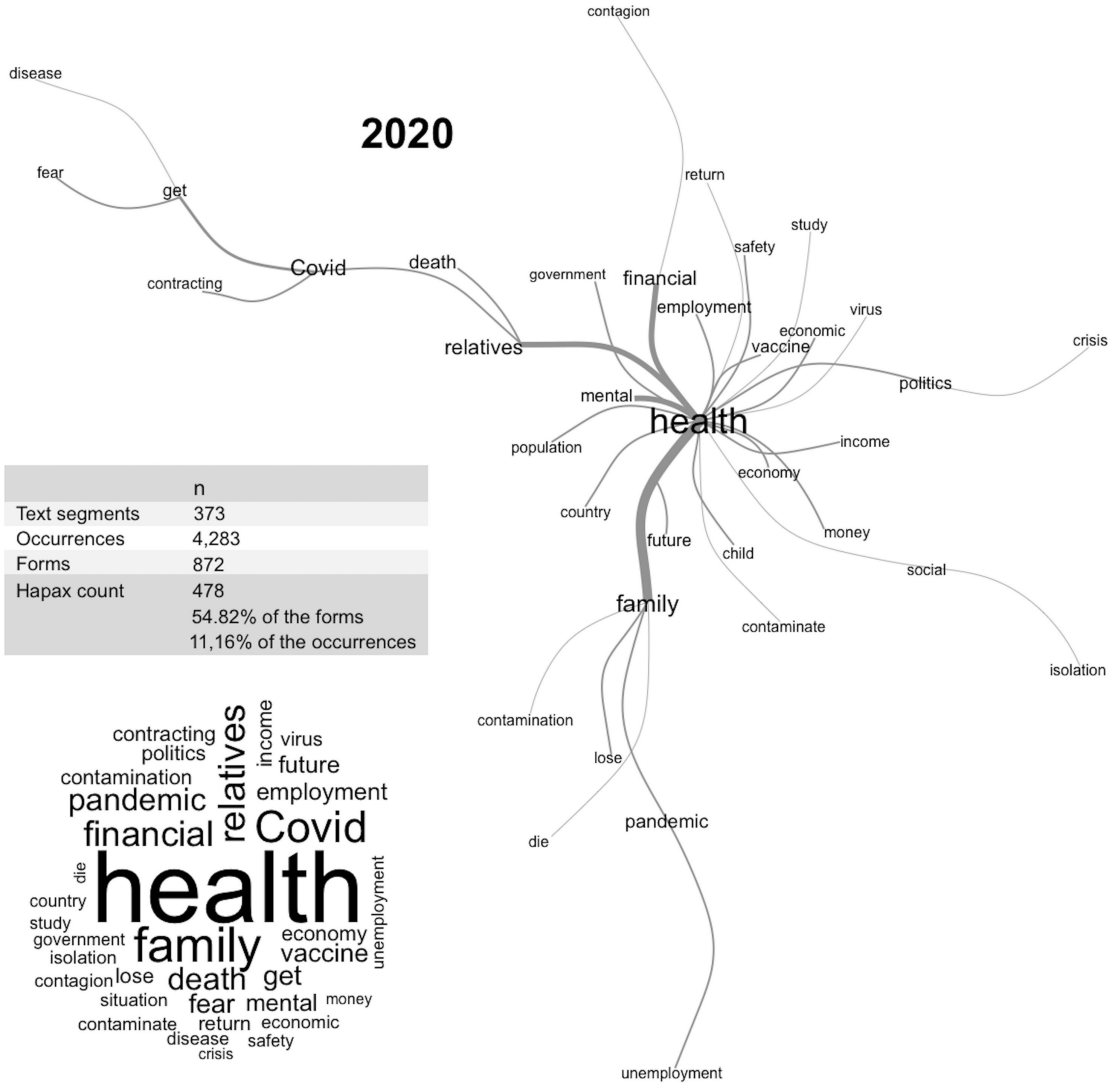
Lexical and similarity analyses of the main concerns of psychologists during the pandemic in 2020.

**Figure 3:**
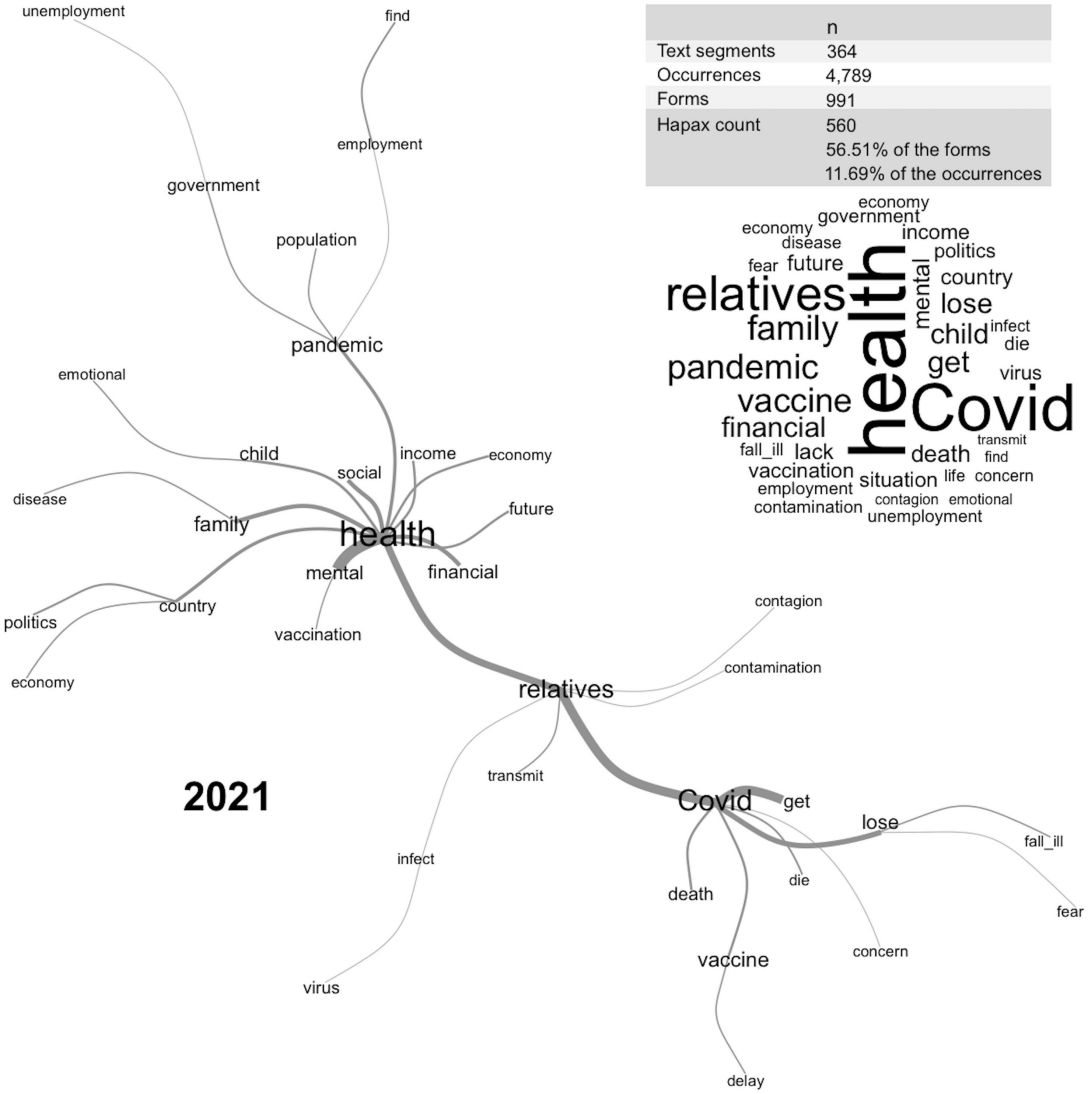
Lexical and similarity analyses of the main concerns of psychologists during the pandemic in 2021.

Figures 2, 3 present the main estimates of the lexical analysis and the diagrams of the similarity analysis, showing the relationship between the main concerns of psychologists in the study phases. In the first year of the pandemic, concerns were clustered on health issues, which branched into two main stems dealing with family concerns. In 2021, three well-defined clusters were identified: ‘health’, ‘family’, and ‘Covid’, with the health cluster branching into general issues ranging from mental health to social issues, employability, and income. The family cluster indicate concerns about the transmission of COVID-19 to family members. The COVID cluster referred to losses, fear, and death.

The prevalence of depression, anxiety, and stress symptoms did not change in the different phases of the study (*z*-test, *p* > 0.05). A high prevalence of at least moderate symptoms was found for depression (~30–40%), anxiety (~25–30%), and stress (~25–30%) (Supplementary Figure 1). Seventy percent of participants had all items of the stress subscale with some degree of impairment. There was a high prevalence of some degree of impairment in items 14 (intolerant) and 18 (touchy), which are part of the depression subscale (Supplementary Table 1).

## Discussion

This study shows that psychologists adopted a remote and hybrid work model with the start of the pandemic that is likely to continue as an alternative for expanding access to mental health care after the pandemic is over. This model of care has been regulated by the Federal Council on Psychology since 2018 (Conselho Federal de Psicologia [CFP], 2018), but with the onset of the pandemic, the regulations were revised to address the immediate needs of the health emergency (Conselho Federal de Psicologia [CFP], 2020). This change expanded the opportunities for professional action to meet a demand that had been growing because of the social isolation imposed as a measure to contain the spread of COVID-19. However, before providing remote care, a psychologist must be able to evaluate the benefits, difficulties, and situations in which this type of care is not feasible. Therefore, training and acquiring the appropriate tools become relevant in order to provide safe, effective, and ethical care (Marasca et al., 2020; Viana, 2020).

A significant proportion of professionals reported that their monthly family income decreased after the onset of the pandemic, which may seem contradictory, given that mental health demands have increased exponentially (Taylor, 2019; Campos et al., 2020; Crepaldi et al., 2020; Faro et al., 2020). However, the political, social, and economical crises in Brazil have negatively affected the income of families in general since the beginning of the pandemic. The Institute for Applied Economic Research has reported a general decrease in household income, affecting mainly the self-employed, which is very common among psychologists (Carvalho, 2021). The report also points out important changes in working hours and absenteeism, which also impacted income (Carvalho, 2021). In this way, inequalities have increased in the Brazilian population, and despite the health crisis, access to mental health care is often limited to the part of the population from higher economic class. However, we can speculate that, given this situation, psychologists might have been forced to lower their fees to allow clients to continue therapy or to facilitate access to new clients. In addition, because we collected data on family income, it is not possible to know the participant’s contribution to decreased income.

Most psychologists indicated that they perceived a change in their mental health since the beginning of the pandemic, which can be explained by the scenario of uncertainty, lack of control, and insecurity (Taylor, 2019; Brooks et al., 2020; Campos et al., 2020; Justo-Henriques, 2020). Feeling of uncertainty was reported by more than 80% of respondents in all phases of the study. This fact can also help us understand the symptoms reported by more than 50% of professionals, such as anxiety, anguish, fear, and insomnia. These characteristics are common in critical situations, where there are sudden changes in routine, unpredictability, and lack of control over both the stressful event and life itself (Taylor, 2019; Brooks et al., 2020; Campos et al., 2020; Faro et al., 2020). However, the findings were somewhat surprising since our sample consisted of mental health professionals and because, despite the knowledge of psychological impact and the presence of symptoms, a considerable number of professionals reported not taking care of their own mental health. Perhaps we can suspect of cognitive dissonance (Festinger, 1962), which is the conflict between beliefs, desires, and values, and should call attention not only to the need for self-care, but also to a thorough evaluation of the reasons why self-care is being neglected. This process should aim at reordering self-awareness and experiences so that the psychotherapist’s stance is consistent with their work with their clients to maintain and/or restore mental health (Harmon-Jones, 2019).

In phase 2 of the study (~9 months after the start of the pandemic, n = 131), an increase in healthy lifestyle behaviors was found, which decreased significantly in phases 3 (n = 378) and 4 (n = 222). To explain this, we can refer to the transtheoretical model proposed by Prochaska (Prochaska and Velicer, 1997; Prochaska, 2018), which presents five phases of behavior change and focuses on the intentionality of change, i.e., the individual’s decision-making process. We can assume that the pandemic acted as a stressor that mobilized internal resources to adapt to the situation, and that in this process some changes were necessary to maintain and/or stabilize people’s physical and mental well-being (pre-contemplation phase). Thus, the change process probably included the need to modify lifestyle by adopting healthier habits (e.g., physical activity and more careful food choices), so that in the first months of the pandemic, people considered behavioral change (contemplation phase), prepared for a change (decision phase), and developed an action plan (action phase). However, with time and the prolongation of the pandemic, the feeling of overload due to the constraints and routine changes may have led to fatigue and the abandonment of the lifestyle changes previously implemented (failure in the maintenance phase), which could explain the significant decrease in the adoption of healthier strategies. Obviously, this reasoning should be taken with caution because the samples in each phase of the study were independent of each other. However, given the large sample size and the monitoring of the mental health of the Brazilian population that we have been conducting since the beginning of the pandemic (Campos et al., 2020, 2021a,b), the use of the transtheoretical model to explain lifestyle changes in the different phases of data collection seems plausible.

Mental health disorders symptoms of the participants were constant across the different phases of the study, suggesting that the strategies used by professionals to care for their own mental health and maintain well-being during the pandemic did not appear to be sufficient. Liao et al. (2014) and Leung et al. (2005) found that affective components have a stronger association with adopting healthier behaviors during a pandemic than cognitive components. Therefore, psychoeducational programs, support groups, and individual therapies for mental health professionals could be considered a priority in the pandemic context.

The concerns about the pandemic reported by psychologists in 2020 were focused on “health” as information about the Sars-Cov-2 virus and COVID-19 was developed and various aspects of life were changed and adjusted around the health crisis. In 2021, with more information and the start of the vaccination program in Brazil, “health” was subdivided into other concerns such as familial transmission of the virus and deaths from COVID-19. This was an expected finding that may be useful in planning support and counseling interventions. These can help psychologists identify connections between their concerns and their symptoms and assess and reassess their strategies and the cognitive, emotional, and social determinants of their behavior to be more effective and better adapt to the effects of the pandemic, now and after.

The study has some limitations, such as the use of a non-probability sample, which makes it difficult to generalize the results to the population of Brazilian psychologists in general. In addition, this was a cross-sectional study, which does not allow for cause and effect associations. However, the present information provides an unprecedented perspective on the mental impact of the pandemic on psychologists, collected at 4 time-points. The sample being mostly women could be another limitation of this study. However, in Brazil, psychology is a profession composed predominantly of women (Bastos and Gondin, 2010; Macedo et al., 2011) and, therefore, our data are close to what is expected in this country. We hope that this study can help further the discussion of mental illness and psychological distress among mental health professionals and support efforts to maintain, restore, and/or recover the well-being of psychologists in the context of the pandemic.

## Conclusion

Psychologists adapted rapidly to the needs of the population and the constraints of the pandemic by shifting to remote and hybrid models of mental health care. However, the pandemic context changed the demand on psychologists and required them to adapt quickly not only in their clinical routine, but also in their personal lives. In this context, a high prevalence of mental health disorders symptoms and difficulties with self-care strategies were observed among psychologists. Thus, actions to raise awareness and promote self-care become important to restore and maintain the health and well-being of psychologists.

## Data availability statement

The raw data supporting the conclusions of this article will be made available by the authors, without undue reservation.

## Ethics statement

The studies involving human participants were reviewed and approved by National Research Ethics Committee of the Ministry of Health (CONEP) (CAAE 30604220.4.0000.0008). The patients/participants provided their written informed consent to participate in this study.

## Author contributions

JuC, LC, BM, and JM: conceptualization and investigation. JuC, LC, BM, AO, FN, SS, JoC, and OP: software. JuC and JM: validation. JuC, BM, LC, and JM: formal analysis. JuC, LC, BM, AO, FN, SS, JoC, OP, and JM: resources. JuC: data curation. LC and BM: writing—original draft preparation. JuC and LC: writing—review and editing. BM, AO, FN, SS, JoC, OP, and JM: visualization. LC: supervision. JuC, OP, and JM: project administration. JuC: funding acquisition. All authors have read and approved the submitted version. All authors have agreed both to be personally accountable for the author’s own contributions and to ensure that questions related to the accuracy or integrity of any part of the work, even ones in which the author was not personally involved, are appropriately investigated, resolved, and the resolution documented in the literature. All authors contributed substantially to the work.

## Funding

This work was supported by grants #2020/08239–6 and #2021/03775–0, São Paulo Research Foundation (FAPESP); and the National Council for Scientific and Technological Development – CNPQ (#303118/2021–0).

## Conflict of interest

The authors declare that the research was conducted in the absence of any commercial or financial relationships that could be construed as a potential conflict of interest.

## Publisher’s note

All claims expressed in this article are solely those of the authors and do not necessarily represent those of their affiliated organizations, or those of the publisher, the editors and the reviewers. Any product that may be evaluated in this article, or claim that may be made by its manufacturer, is not guaranteed or endorsed by the publisher.

## Footnotes

^1^Phase 1: May 18 to June 23, 2020, COVID-19 total cases: 1,228,114; total deaths: 54,971; average cases/day: 25,308.6; average deaths/day: 996.2; Phase 2: November 18 to December 23, 2020, total cases: 7,448,560; total deaths: 190,488; average cases/day: 40,442.2; average deaths/day: 626.0; Phase 3: May 18 to June 23, 2021, total cases: 18,322,760; total deaths: 511,142, average cases/day: 68,342.8; average deaths/day: 1,912.9; Phase 4: November 18 to December 23, 2021, total cases: 22,234,626; total deaths: 618,424; average cases/day: 6,762.2, average deaths/day: 173.0.

## Supplementary material

Supplementary Figure 1 Distribution of professionals according to the degree of involvement by symptoms of depression, anxiety and stress.
