# Supplementary material for: COVID-19 pandemic: Prevalence of depression, anxiety, and stress symptoms among Brazilian psychologists
Source: Front Psychol. 2022 Dec 2;13:1012543. doi: 10.3389/fpsyg.2022.1012543 (PMC9756808; doi:10.3389/fpsyg.2022.1012543)
Supplement: Supplementary file 2 [file Table_1.docx]

**Supplementary Table 1.** Distribution of professionals according to the responses to the Depression, Anxiety and Stress Scale (DASS-21).

|  | **Response - n(%)** | | | |
| --- | --- | --- | --- | --- |
| **item** | **never** | **sometimes** | **very often** | **almost always** |
| it1. ...hard to wind down | 281 (28.3) | 579 (58.3) | 117 (11.8) | 16 (1.6) |
| it2. ...dryness of mouth | 561 (56.4) | 309 (31.1) | 82 (8.2) | 41 (4.1) |
| it3. ...no positive feeling... | 404 (40.7) | 453 (45.7) | 121 (12.2) | 14 (1.4) |
| It4. ... breathing difficulty... | 624 (62.8) | 306 (30.8) | 55 (5.5) | 6 (0.6) |
| It5. ...difficulty to work up the initiative... | 220 (22.2) | 467 (47.0) | 229 (23.1) | 77 (7.8) |
| It6. ...over-react to situations | 287 (28.9) | 534 (53.8) | 154 (15.5) | 8 (1.8) |
| It7. ...trembling... | 776 (78.1) | 174 (17.5) | 35 (3.5) | 8 (0.8) |
| It8. ...a lot of nervous energy... | 285 (28.7) | 518 (52.1) | 161 (16.2) | 29 (2.9) |
| It9. ... panic and make a fool of myself | 679 (68.3) | 233 (23.4) | 67 (6.7) | 14 (1.4) |
| it10. ... nothing to look forward to | 542 (54.6) | 309 (31.1) | 90 (9.1) | 52 (5.2) |
| It11. ...getting agitated | 200 (20.2) | 536 (53.9) | 218 (21.9) | 38 (3.8) |
| It12. ...difficult to relax | 128 (12.9) | 522 (52.5) | 260 (26.2) | 83 (8.4) |
| it13. ...down-hearted and blue | 195 (19.6) | 534 (53.7) | 192 (19.3) | 72 (7.2) |
| It14. I was intolerant... | 333 (33.5) | 517 (52.1) | 120 (12.1) | 23 (2.3) |
| It15. ...close to panic... | 744 (74.9) | 205 (20.6) | 37 (3.7) | 7 (0.7) |
| it16. I was unable to become enthusiastic... | 433 (43.6) | 410 (41.3) | 104 (10.5) | 46 (4.6) |
| It17. ...I wasn't worth much... | 564 (56.9) | 287 (28.9) | 88 (8.9) | 53 (5.3) |
| It18. ...I was rather touchy | 71 (7.1) | 466 (46.9) | 342 (34.4) | 114 (11.5) |
| it19. I was aware of the action of my heart... | 536 (53.9) | 311 (31.3) | 95 (9.6) | 51 (5.1) |
| it20. I felt scared... | 598 (60.2) | 321 (32.3) | 63 (6.3) | 11 (1.1) |
| It21....life was meaningless | 541 (54.4) | 298 (30.0) | 107 (10.8) | 47 (4.7) |
